# Leveraging data to support health equity in an integrated delivery and finance system

**DOI:** 10.1002/lrh2.10423

**Published:** 2024-04-15

**Authors:** Jane Kogan, Joan Eichner, Hyagriv Simhan, Erin Dalton, Alex Jutca, Beth Quinn, Jennifer Chaney, Anna Patterson, Donna Keyser

**Affiliations:** ^1^ UPMC Insurance Services Division and UPMC Center for High‐Value Health Care Pittsburgh Pennsylvania USA; ^2^ UPMC Insurance Services Division and UPMC Center for Social Impact Pittsburgh Pennsylvania USA; ^3^ Department of Obstetrics, Gynecology and Reproductive Sciences University of Pittsburgh School of Medicine Pittsburgh Pennsylvania USA; ^4^ UPMC Magee Womens Hospital Pittsburgh Pennsylvania USA; ^5^ Allegheny County Department of Human Services Pittsburgh Pennsylvania USA

**Keywords:** data integration, health equity, SDOH

## Abstract

**Introduction:**

To accelerate healthcare transformation and advance health equity, scientists in learning health systems (LHSs) require ready access to integrated, comprehensive data that includes information on social determinants of health (SDOH).

**Methods:**

We describe how an integrated delivery and finance system leveraged its learning ecosystem to advance health equity through (a) a cross‐sector initiative to integrate healthcare and human services data for better meeting clients' holistic needs and (b) a system‐level initiative to collect and use patient‐reported SDOH data for connecting patients to needed resources.

**Results:**

Through these initiatives, we strengthened our health system's capacity to meet diverse patient needs, address health disparities, and improve health outcomes. By sharing and integrating healthcare and human services data, we identified 281 000 Shared Services Clients and enhanced care management for 100 adult Medicaid/Special Needs Plan members. Over a 1‐year period, we screened 9173 (37%) patients across UPMC's Women's Health Services Line and connected over 700 individuals to social services and supports.

**Conclusions:**

Opportunities exist for LHSs to improve, expand, and sustain their innovative data practices. As learnings continue to emerge, LHSs will be well positioned to accelerate healthcare transformation and advance health equity.

## 1 INTRODUCTION

Ongoing efforts to harness the value of big data coupled with increased interest in the learning health system (LHS) model offer important opportunities to accelerate healthcare transformation and advance health equity.^1^, ^2^, ^3^, ^4^, ^5^ National calls for health systems to account for and mitigate the negative impact of social factors that influence health, interchangeably referred to as social determinants of health (SDOH),^6^ invariably focus on the need for innovative approaches to overcome common data challenges, such as more consistent and reliable collection and use of SDOH data as well as cross‐sector and within system operability of data.^7^, ^8^, ^9^, ^10^, ^11^, ^12^ Since data available within healthcare systems and county or state health and human services departments have been historically managed separately and are defined in different ways across settings,^13^, ^14^, ^15^ the interoperability issues are often considered to be insurmountable. Additional barriers to cross‐sector data sharing include healthcare privacy laws and regulations,^16^ which are not well defined and vary across states,^17^, ^18^, ^19^, ^20^, ^21^ and the lack of a robust data infrastructure to safely transmit and store the data. System‐level challenges are also well documented^22^ and include provider incentives to screen for patients' social needs but not necessarily connect them to available social services, limited and/or unpredictable staff resources within healthcare systems to make these connections, siloed provision of healthcare and human services hindering providers' awareness of a patient's social service use, including completion of referrals, and administrative burden (eg, repeated phone calls, adjustments to workflows).^18^, ^19^, ^23^ Patients may also be reluctant or unwilling to self‐report social risk factors and/or admit their need for social services and supports.^22^, ^24^

Despite these hurdles, progress is being made.^10^, ^12^, ^25^, ^26^, ^27^, ^28^, ^29^, ^30^ Some LHSs are working to integrate cross‐sector data to better meet patients' holistic needs, and others are looking internally to identify where data capture, workflow, and connection of patients to needed resources can be improved. UPMC, a nonprofit academic medical center and one of the nation's leading integrated delivery and finance systems (IDFS), is leveraging its robust learning ecosystem to make headway on both fronts, reflecting its institutional commitment to advance health equity by serving patients, groups, and communities that experience disadvantage and disparities in health outcomes. In this paper, we describe two UPMC initiatives and their results, share lessons learned, and offer insights on future directions for other LHSs seeking to enhance their data practices.

## 2 CROSS‐SECTOR INITIATIVE TO INTEGRATE HEALTHCARE AND HUMAN SERVICES DATA FOR BETTER MEETING SHARED SERVICES CLIENTS' HOLISTIC NEEDS

### 2.1 Setting and context

UPMC has its headquarters in Pittsburgh, Pennsylvania, where the Allegheny County Department of Human Services (ACDHS) directly provides or contractually administers a breadth of services, such family strengthening and youth programs, child protective services, homeless services, behavioral health services, supports for people with intellectual disabilities/autism and older adults, jail re‐entry programs, crisis supports, and violence prevention, for 1.2 million county residents. As the largest payer and Medicaid managed care organization in the region, UPMC Health Plan insures approximately 60% of these residents. Given the significant overlap of our populations and the comprehensive, integrated data warehouses and sophisticated analytic work^31^ of each organization, UPMC Health Plan and ACDHS embarked on a cross‐sector effort to integrate healthcare and human services data. Our goal was to provide more holistic, efficient, and equitable care for our Shared Services Clients (ie, individuals who are enrolled in any UPMC Health Plan insurance product and receive ACDHS services and supports).

### 2.2 Approach

In 2018, UPMC Health Plan initiated steps to enter into a formal data use agreement (DUA) with ACDHS. Depending on the circumstances and scope of services delivered to the Shared Services Clients, UPMC Health Plan and ACDHS operate as either Covered Entities or Business Associates under the Health Insurance Portability and Accountability Act (HIPAA). The DUA negotiations spanned issues related to privacy, business risks, technical requirements, and applicable laws and regulations. Under the DUA, UPMC Health Plan and ACDHS can “exchange healthcare and human service utilization information to inform the treatment, care coordination, operational and programmatic coordination, and facilitation of social services for shared clients, and guide quality assurance efforts to improve the delivery of care.” UPMC Health Plan consulted with internal legal, data governance, and privacy experts to determine what minimally necessary data elements could be shared securely. Specially protected or sensitive information/data, such as substance use history or HIV/AIDS status, is not covered by the agreement so these and similar data are not shared. Both organizations mutually created and standardized a data sharing model that aligns with “Treatment, Payment, and Health Care Operations” regulations of the HIPAA Privacy Rule.

**FIGURE 1:**
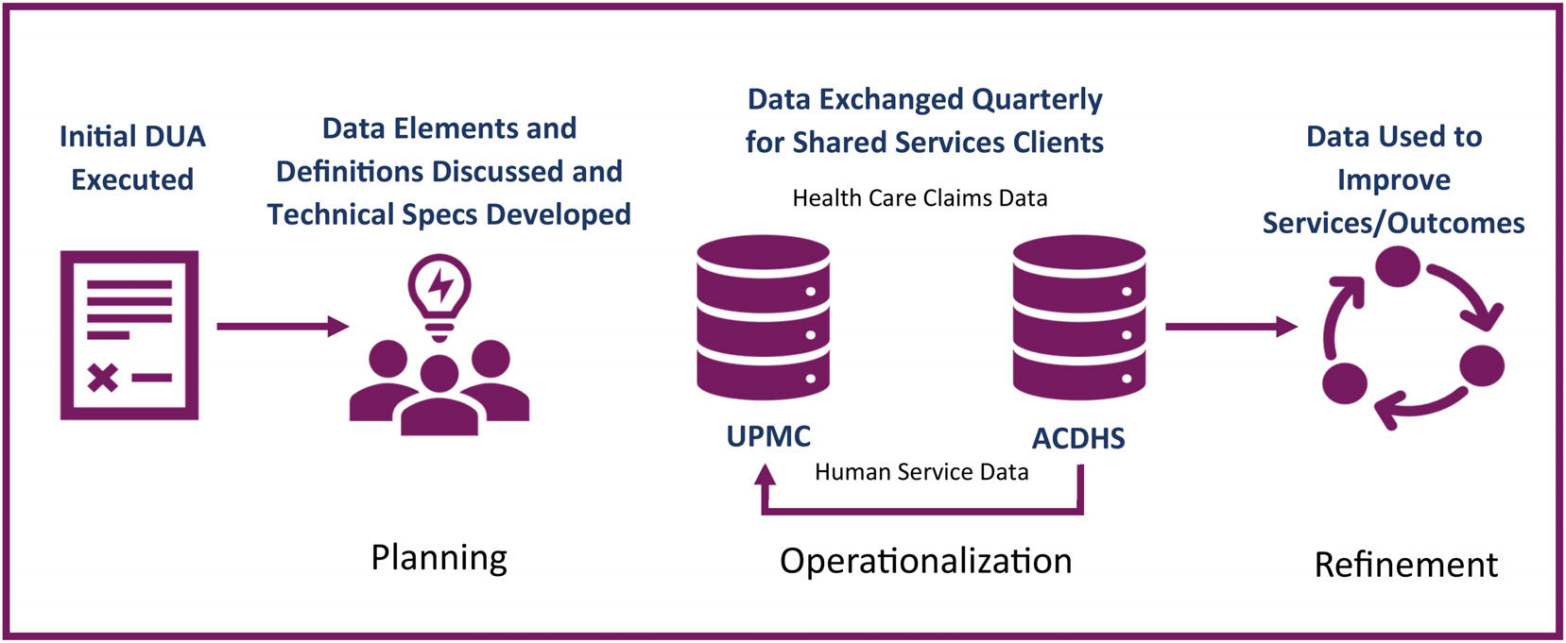
Cross‐sector data harmonization process. ACDHS, Allegheny County Department of Human Services; DUA, data use agreement.

Once the DUA was fully executed, monthly meetings were held with UPMC Health Plan and ACDHS staff, including subject matter experts on information technology, data governance, legal, privacy/HIPAA, compliance, analytics, and research, to develop a data sharing process and associated procedures (Figure 1). The team worked together to establish a secure electronic file transfer site between the organizations and detailed specifications for the data exchange, including master ID numbers, timing and frequency of the exchange, and balancing the need for useful data exchange, while still meeting the necessary standards within HIPAA. Over a 1‐year period, the technical details of the exchange were tested and refined, including file creation, electronic transfer, automation, and data interpretation. Data structure, definitions, and interpretation had to be clearly defined, often requiring significant discussion, before data could be meaningfully utilized by each entity.

Quarterly, UPMC Health Plan now generates and sends to ACDHS an automated electronic data file containing current, fully insured members of all insurance products who reside in Allegheny County. Upon receipt of the file, ACDHS matches the UPMC Health Plan member list with active human service clients and returns to UPMC Health Plan the Shared Services Clients list for that quarter and requested human service data for individuals on the list. The dataset indicates active enrollment in each type of human service program and enrollment dates as well as service provider agency and service coordination information when applicable. UPMC Health Plan sends ACDHS an automated electronic data file containing demographics, contact information, and the requested healthcare utilization data on all Shared Services Clients. The dataset includes type and date of recent healthcare utilization, primary diagnosis associated with each encounter, care management participation, and contact information when applicable. The original membership file that was exchanged in December 2019 covered January 2017 through November 2019. Ongoing, the active period of data exchange is defined as the time from 2 years prior through the current date.

### 2.3 Results

**FIGURE 2:**
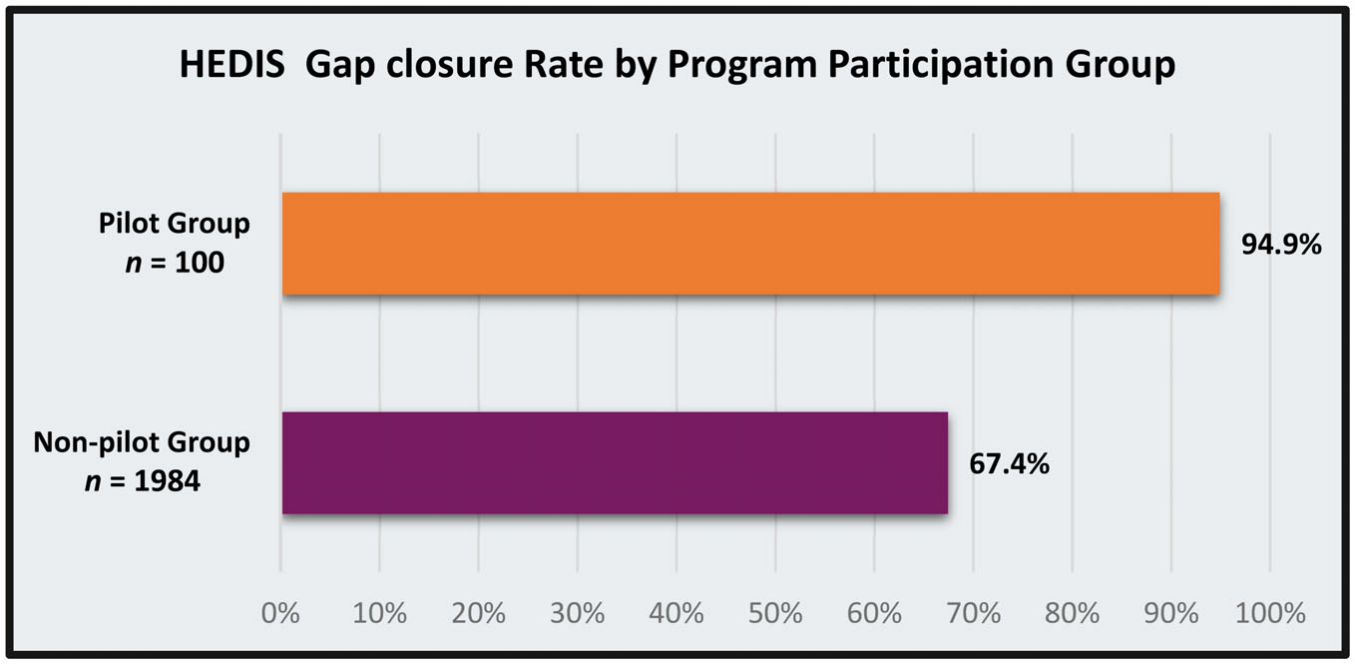
HEDIS gap closure rate.

**FIGURE 3:**
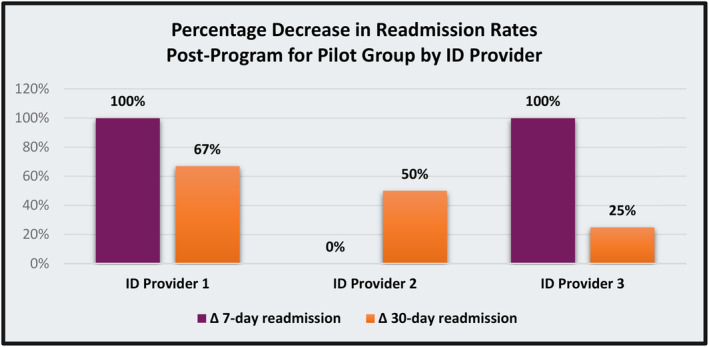
Pilot group post‐program readmission rates.

The original membership file exchange in December 2019 identified over 281 000 Shared Services Clients. Since that time, the volume of shared clients has increased by approximately 25 000 each year and, at end of 2023, the membership file included 385 844 individuals. Due to this high‐volume of data integration at the county level, UPMC Health Plan has been able to enhance care coordination services for UPMC Health Plan members in numerous ways. A case in point is a newly acquired capacity to identify adult Medicaid/Special Needs Plan members with intellectual and development disabilities or autism (ID/A). Healthcare claims data do not indicate if a member has an ID/A diagnosis or provide essential information about their daily living environment, such as residence in a group facility. In such cases, ID/A service providers are intimately knowledgeable about and involved in members' lives. For example, they often manage members' daily physical health needs and coordinate access to healthcare. However, the members' health insurer has no information about the ID/A service provider. Data integration with ACDHS enabled UPMC Health Plan to identify ID/A providers, services, and waiver type for each member and receive information about their diagnosis. Using this information, UPMC Health Plan established an Enhanced Care Management Pilot (ECMP) involving health plan care managers and three intellectual disability service providers caring for our members. For the first group of ECMP members (n = 100), we observed a nearly 95% gap closure rate for HEDIS measures for program participants as compared to a 67% for those not enrolled in ECMP (n = 1984) (Figure 2). Additionally, for members who were in ECMP, after 12 months and across three providers, we observed an overall 50% reduction in 7‐ and 30‐day hospital readmissions (Figure 3), 100% completion rate for annual health risk assessments. We note that these outcomes represent pre‐post comparisons and, due to limited sample sizes and incompleteness of data, no statistical testing was performed. As such, interpretation is subject to common limitations inherent with observed trends, such as possible regression to the mean. Finally, we observed improvements in routine communication between plan‐based care managers and IDD providers, a process feature that was not occurring prior to data integration, which includes sharing critical information about coverage allowances, such as over‐the‐counter medical supplies and durable medical equipment.

## 3 SYSTEM‐LEVEL INITIATIVE TO COLLECT AND USE PATIENT‐REPORTED SOCIAL NEEDS DATA FOR CONNECTING PATIENTS TO RESOURCES

### 3.1 Setting and context

UPMC's Women's Health Service Line (WHSL) comprises 15 birthing hospitals, including large academic medical facilities, critical access centers, and rural hospital settings across Pennsylvania, New York, and Maryland. Collectively 25 000 babies are delivered at these hospitals each year. The patient population is diverse with a substantial portion considered high risk for poor social, physical, and mental health outcomes. Since most patients seek care through UPMC employed providers, we implemented a standardized process for screening and monitoring patient risk factors and allocating resources and educational support. Nonetheless, given the amount of educational information to be shared during each office visit, it is often not possible to adequately screen patients and document all relevant social risk factors which, if not detected until later, can have negative impacts on health outcomes for both mothers and babies.

Until late 2022, WHSL's OBGYN offices utilized a paper‐based, homegrown psychosocial risk assessment to capture and document information related to patients' social needs. The screening tool was not comprehensive, and the process for documenting and following up on actions to address patients' needs in the EHR was suboptimal. These gaps led to an OB service‐wide commitment to address inconsistencies in capturing SDOH data along with plans to improve the WHSL resource bandwidth for addressing social needs.

### 3.2 Approach

At the end of 2022, we began implementing the Epic Social Factors screening tool^32^ as a standardized measure to capture information on patients' SDOH through the EHR in outpatient settings across the WHSL. This tool categorizes social risk factors within nine SDOH domains, namely physical activity, financial resource strain, housing stability, transportation needs, food insecurity, stress, social connections, intimate partner violence, and alcohol use. It produces a risk categorization based on 23 questions with various patient response options. Patients complete the screening via MyUPMC, a patient portal, or on an electronic tablet upon registration for their first prenatal visit. Responses are auto recorded in the patient's electronic chart and incorporated into an SDOH summary in the EHR.

**FIGURE 4:**
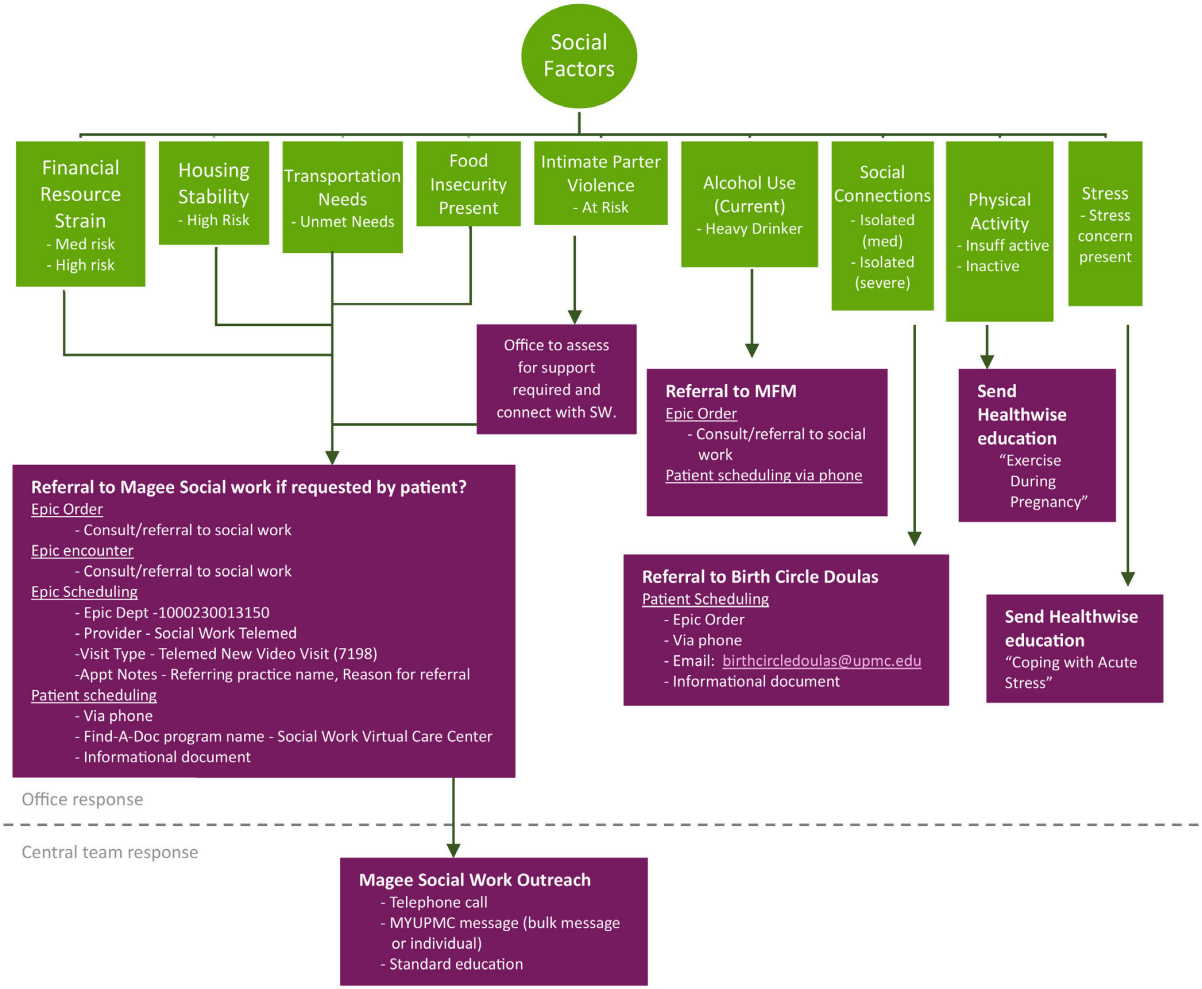
Women's Health Service Line social needs screening and referral pathway. MFM, maternal fetal medicine; MyUPMC, name of the UPMC patient portal; SW, social worker.

To enhance the usability of this information, we partnered with UPMC Magee‐Womens Hospital social work and UPMC's analytics teams to design and build a population health tool (or electronic worklist) for managing patients' SDOH data. As part of this process, we aligned the tool's functionality with WHSL population health management approaches for connecting patients with essential clinical services while also ensuring that it provided discrete information on SDOH needs and recommended actions based on a standard pathway for resource referral (Figure 4). This work was led by the WHSL's Clinical Innovation Team, in collaboration with colleagues in outpatient OBGYN offices, hospital social work, Epic, and UPMC Clinical Analytics. Over a 6‐month period, the group worked together to gain full understanding of the Epic Social Factors tool and its impact on existing screening tools, design a standard screening and referral pathway, and implement the screening process with Epic in outpatient offices. To provide additional guidance around connecting patients to needed resources, we created and distributed informational resources to all OBGYN offices in the WHSL. The tool is now used by a WHSL centralized care team of population health nurses and navigators who document actions taken for SDOH needs in the patient's chart and ultimately enabling the identification of gaps closed over time.

### 3.3 Results

Over a 1‐year period, 9173 actively pregnant patients or nearly 37% of individuals served in the WHSL completed the Epic Social Factors screening tool. Among the nine domains of the tool, the frequency of positivity was highest for insufficient physical activity and social isolation followed by mental health and financial strain. For the five domains with the highest frequency of positivity, nearly 10% of patients reported positivity in two or more domains (Table 1). Over the same period, integration of social data into an electronic worklist enabled the WHSL Social Work team to follow‐up on over 700 referrals for high‐risk patients who reported two or more needs. Since the roll‐out of Epic Social Factors screening in OB/GYN offices, a standard process for engaging with each patient via phone or a scheduled visit has been established. Once connected, the team shares resources via UPMC's patient‐facing electronic portal facilitating access to social services and supports and providing education and reminders on preventive health services and routine screenings. The revised workflow also includes an integrated population health data dashboard that provides the Social Work team with real‐time information on individual patient‐level gaps in care to assist with gap closure. The development and implementation of this dashboard occurred over a 6‐month period and included consideration of the underlying dashboard logic, validation with expert teams responding to potential risks identified, and rollout of the approach with a central team and outpatient offices. Beginning in fall 2023, the central team began notifying patients and/or patients' providers as gaps for patients not yet screened and risks associated with substance use were identified. With the central team's hiring of a dedicated social worker, the OB/GYN social work team also began responding to Epic referrals placed by outpatient offices from across the state. The subsequent and ongoing phase, initiated in early 2024, comprises central team use of results from the screening tool and information from the population health worklist to close gaps for patients with risks in five key domains, namely financial resource strain, housing instability, transportation needs, food insecurity, and intimate partner violence.

**TABLE 1:** Rates of positive screening and identification of social needs.

Social determinates of health domains	
Domain	Frequency of positivity
Financial resource strain	10.2%
Housing instability	8.2%
Unmet transportation needs	4.9%
Food insecurity	9%
Intimate partner violence	3.6%
Heavy alcohol use	6%
Moderate–Severe social isolation	34.7%
Insufficient physical activity	50.8%
High psychological stress	15.7%

## 4 LESSONS LEARNED

As these two initiatives demonstrate, by integrating healthcare and social/human services data, UPMC has improved its capacity to understand and meet diverse patient and member needs, address health disparities, and improve health outcomes. Not surprisingly, we encountered many of the same barriers commonly reported in the literature related to these types of innovative data practices. In Table 2, we offer three basic strategies and associated tactics that stakeholders might consider for overcoming these barriers as they work to advance their own healthcare transformation and health equity goals.

**TABLE 2:** Strategies and tactics to overcome barriers in data integration.

Strategy and tactics in data integration practices	
Strategy	Practical tactics
Build trusting relationships	Take time to develop rapport among all stakeholder groups Develop shared understanding and terminology for privacy and interoperability issues Balance innovation with business risks Understand and enforce compliance with applicable laws and ensure protections are in place Set clear expectations of data governance and stewardship
Utilize a multidisciplinary approach	Leverage existing data governance committees Align data functionality with other system‐wide procedures Build cross‐departmental or organizational technical teams Develop flexible and modifiable data‐sharing templates Clearly articulate and revisit the business value for all stakeholder entities
Make upfront investments for downstream return	Consider resources required for all stages of the initiative Provide upfront staff time to engage in the design so plans are well informed and practical Test implementation plans and prepare to adjust Make reasonable estimates and plans for the necessary resources to adjust, grow, and sustain new practices

Consideration and use of these strategies and tactics can ease the process and quicken the pace toward effective changes in SDOH data practices. For example, in the UPMC Health Plan and ACDHS cross‐sector data sharing initiative, initial establishment of data specifications and transfer procedures required considerable time and effort, as did learning how to manage and interpret each sector's data. While both sides were highly data savvy, many questions arose about how data are formatted and their meanings. The sheer size of the data files created manageability issues as well. Working cross‐sector presented differences in terminology and varying levels of familiarity with HIPAA regulations. To address these issues, UPMC Health Plan and ACDHS maintained a monthly leadership touchpoint where challenges could be discussed. Over time, leaders made additions and modifications to the composition and formatting of the data files, internal workflows, and data interpretation. As the partnership matured and technical details were resolved, we organized small workgroups to focus on operationalizing the data around specific use cases. With respect to the system‐level WHSL initiative, considerable upfront staff time was required to design and incorporate the screening and population health management tools into the EHR. However, ongoing resources were also required during the implementation stage. Following standardized use of the screening tool at WHSL's OBGYN offices, almost 40% of the 25 000 women served in these practices each year were identified as having social needs. This means 65 women a day, in this one service line alone, needed to be connected to social services and/or other resources. Most, if not all, of these connections did not happen with a press of a button or a single email/phone call. If resource allocation and/or provider bandwidth are limited once a screening tool is implemented, the needs will outweigh the resource, and health systems may continue to find social factors negatively affecting physical and mental health outcomes.

## 5 FUTURE DIRECTIONS

UPMC's integrated LHS model is rapidly gaining experience and momentum with innovative data practices for accelerating our pace to accelerate healthcare transformation and advance health equity. At present, we are considering ways to sustain and scale the two initiatives described above. At the cross‐sector level, we hope to replicate the data exchange model between UPMC Health Plan and ACDHS with human services departments in other Pennsylvania counties that serve large numbers of our members or even statewide through a DUA with the Pennsylvania Department of Human Services. However, given the variable maturity of county‐level data resources across the Commonwealth, using this model to improve care coordination for health plan members outside of Allegheny County will require a high level of interest, commitment, and flexibility from all key stakeholders. Ideally, at the system level, we will be able to incorporate the WHSL screening and population health management tools into other service line operations where a substantial portion of patients are at high risk for poor social, physical, and mental health outcomes. Moreover, building on the two innovative data practices described here, our integrated LHS is actively pursuing the combination of human services and healthcare delivery (ie, EHR and claims/administrative) data as a more complete, and thus more actionable, strategy to provide more holistic, efficient, and equitable care for the individuals we serve.

We also see considerable opportunity for LHSs to address SDOH and advance health equity through closer partnerships with a wide range of community‐based organizations (CBOs). Although many of these organizations have limited information technology capabilities, data infrastructure, and knowledge of regulatory requirements for sharing protected health information, we remain optimistic about future collaborations for improving the health of the individuals and communities we serve. The recent request for Information from the Office of Civil Rights to reform key HIPAA statutes to better define privacy laws and remove or reduce obstacles that currently hinder the ability to share data between covered entities (ie, hospitals) and third parties^33^ (ie, CBOs) fuels this optimism. As more federal and state agencies heed the call for privacy reform, we can easily imagine a day when all organizations will be able to exchange or view records of Shared Services Clients.

In the meantime, UPMC Health Plan continues to develop new use cases for healthcare and human services data integration, for example, identifying opportunities to collaborate with CBOs that address health‐related social needs and operate in communities where there is a documented need to reduce health inequities experienced disproportionally among members of color, who often have high rates of human service involvement compared to the general population. For example, UPMC Health Plan has funded CBOs to employ community health workers who outreach to members in home and community settings to assess and refer for social needs, provide public benefits enrollment assistance, and support members to close open gaps in care such as annual primary care appointments and dental checkups. Integrated data allow the plan to look at households in the geographic service area of these CBOs and understand the complete health and human service picture of the household members. As key players in the national LHS, we can serve as champions of forthcoming Medicaid requirements to collect SDOH data, accelerate the move to managed care for ID/A services, and ensure that CMS and state Medicaid screening measures for social risk factors are incorporated into value‐based payment models.

## CONFLICT OF INTEREST STATEMENT

Jane Kogan, Joan Eichner, Donna Keyser, Beth Quinn, and Jennifer Chaney are employees of UPMC, the learning health system that was involved/leading the activities described in this manuscript. Hy Simhan is an employee of UPMC and University of Pittsburgh, Consultant to Organon, Authorship with honorarium for UpToDate, and Cofounder of Naima Health, LLC. Erin Dalton and Alex Jutca are employees of Allegheny County Department of Human Services, an entity that was involved/leading some of the activities described in this manuscript. Anna Patterson was previously an employee of UPMC and has no conflicts to report.
